# Optimization of Plant Oxalate Quantification and Generation of Low-Oxalate Maize (*Zea mays* L.) through *O7* Overexpression

**DOI:** 10.3390/plants13212950

**Published:** 2024-10-22

**Authors:** Kai Zhao, Tao Wang, Bin-Bin Zhao, Jun Yang

**Affiliations:** 1 National Engineering Laboratory of Crop Stress Resistance, College of Life Science, Anhui Agricultural University, Hefei 230036, China; zhaokai@stu.ahau.edu.cn (K.Z.); zhaobinbin@ahau.edu.cn (B.-B.Z.); 2 Department of Biology and Food Engineering, Bozhou University, Bozhou 236800, China; 2022110014@bzuu.edu.cn

**Keywords:** oxalate, UPLC-QqQ-MS/MS, oxalyl-CoA synthetase, overexpression, agronomic traits

## Abstract

Oxalate, the simplest dicarboxylic acid, is a prevalent antinutrient that chelates with various metals and can lead to the formation of kidney stones in humans. The accurate detection of the oxalate concentration in food and the cultivation of low-oxalate crops are important for enhancing public health. In this study, we established a high-throughput and highly sensitive technique for oxalate detection using ultra-high-performance liquid chromatographic–triple quadrupole tandem mass spectrometry (UPLC-QqQ-MS/MS). Additionally, we overexpressed the gene *O7*, which encodes oxalyl-CoA synthetase in the maize oxalate degradation pathway, resulting in *O7*-OE lines. By employing the UPLC-QqQ-MS/MS method to measure oxalate levels in these transgenic lines, we observed that the oxalate content in the kernels of *O7*-OE lines was reduced by approximately 43%, with a concurrent increase in some micronutrients such as zinc. Importantly, the transgenic maize showed normal seed storage compound accumulation or other agronomic characteristics. In summary, we developed a high-throughput detection method that advances oxalate measurement. Furthermore, by generating new maize germplasm with diminished oxalate, our work offers potential health advantages to consumers.

## 1. Introduction

Oxalate, a dicarboxylic acid and prevalent antinutritional factor, is widely found in certain plant species. Due to its acidic properties and strong ability to chelate, oxalate often forms insoluble salts when combined with calcium, magnesium, or iron, whereas it forms soluble complexes with sodium, potassium, or ammonium [1]. These two forms of oxalate are predominant in plant tissues. Oxalate serves a beneficial role in plants by helping them adapt to stressful conditions. However, oxalate in food can interfere with the absorption and utilization of vital nutrients in the human body, such as calcium, zinc, and copper [2,3]. Notably, numerous studies have demonstrated a strong association between oxalate and the formation of kidney stones in humans. Approximately 80% of the most common types of kidney stones are composed of oxalate calcium [4]. Hence, the precise detection of oxalate concentrations in food and the development of low-oxalate food products are crucial for improving public health by mitigating the potential adverse effects associated with high oxalate intake. This aligns with the requirements for future smart crop design [5].

Currently, various methods are utilized for detecting oxalate content, including enzymatic assays using oxalate oxidase [6,7], high-performance liquid chromatography (HPLC) [8,9], capillary zone electrophoresis (CZE) with conductivity detection [10], gas chromatography–mass spectrometry (GC-MS) [11], amperometry [12], ion chromatography (IC) with chemiluminescence detection [13], and headspace gas chromatography (HS-GC) [14]. Each of these techniques has its own set of strengths and limitations. Among them, HPLC is the most commonly used for oxalate detection, favored for its high sensitivity, accuracy, and robustness [15,16,17]. The typical detectors used with HPLC are ultraviolet (UV) detectors. The HPLC-UV detection system provides excellent detection accuracy, particularly when analyzing samples with high oxalate content and a single type of compound. However, in complex sample matrices where oxalate levels are low and various compounds are present, HPLC-UV may not always yield accurate detection. The presence of interfering compounds can complicate the accurate quantification of oxalate in unrefined sample preparations [18].

Hydrophilic interaction liquid chromatography (HILIC) operates with an elution opposite to that of reverse-phase (RP) chromatography, resembling normal-phase chromatography [19,20]. In HILIC, gradient elution typically begins with low-polarity organic solvents and progresses to higher water content to elute polar analytes [21,22]. This unique feature allows HILIC to effectively separate compounds that have traditionally been challenging to analyze, including small organic acids, polar drugs, and many neutrally charged substances. Oxalate, characterized by its two hydrophilic carboxyl groups, exhibits high polarity. In contrast to conventional RP-LC (reverse-phase liquid chromatography), HILIC enhances the retention of oxalate due to its affinity for polar interactions, thereby improving detection accuracy. Furthermore, the buffer salts used in HILIC are compatible with mass spectrometry (MS), making it especially suitable for combined techniques where chromatographic separation is coupled with MS detection. This compatibility broadens the application of HILIC to include the quantitative analysis of polar compounds with the sensitive and selective detection capabilities provided by MS [21].

The multiple reaction monitoring (MRM) mode of triple quadrupole tandem mass spectrometry (QqQ-MS/MS) is a fast and highly sensitive analytical technique used for the selective detection and quantification of phytochemicals. By scanning specific precursor ions and generating product ion transitions, MRM significantly reduces analysis time and eliminates concerns about interference from overlapping peaks [23,24]. This method offers exceptional sensitivity with extremely low limits of quantitation. Combining the high sensitivity and specificity of QqQ-MS/MS, an ultra-high-performance liquid chromatograph coupled with a triple quadrupole tandem mass spectrometer (UPLC-QqQ-MS/MS) based on HILIC is currently considered an optimal approach for detecting oxalate in unrefined sample preparations. HILIC’s capability to retain polar compounds, such as oxalate, enhances the detection efficiency of the method. Developing a high-throughput and highly sensitive technique for oxalate detection is crucial for efficiently assessing its levels in food through methods like breeding or genetic modification.

Overexpression of key enzymes involved in oxalate degradation can effectively reduce oxalate content. The heterologous overexpression of oxalate oxidase significantly lowered the oxalate levels and enhanced the resistance of transgenic plants to oxalate [25,26,27]. Similarly, the overexpression of the *AAE3* gene, which encodes acyl-activating enzyme 3 and catalyzes the conversion of oxalate to oxalyl-CoA (the first step in the oxalate degradation pathway), has proven effective in reducing oxalate content in transgenic Arabidopsis plants [28]. Deficiencies in crucial enzymes involved in maize oxalate degradation, namely oxalyl-CoA synthetase (O7, analogous to AAE3) and oxalyl-CoA decarboxylase1 (OCD1, responsible for converting oxalyl-CoA to formyl-CoA in the second step), lead to significant oxalate accumulation and an opaque endosperm in their mutant kernels [29,30]. However, it remains uncertain whether genetic engineering approaches targeting oxalate degradation enzymes can reduce the oxalate content in crops. We are exploring the feasibility of genetically modifying maize to lower oxalate levels by targeting O7 and OCD1 enzymes.

In this study, we optimized a UPLC-QqQ-MS/MS detection system for oxalate to measure its levels in plants. We then employed a constitutive promoter-driven overexpression of maize oxalate degradation genes *O7* and *Ocd1* to assess their ability to reduce oxalate content in kernels. We found that the overexpression of *O7* does not affect ear development, kernel performance, or the accumulation of storage substances. Notably, there is a significant decrease in the oxalate content within the kernels. Additionally, the overexpression of *O7* resulted in a concurrent increase in micronutrients in the kernels.

## 2. Results

### 2.1. Oxalate Quantification Using UPLC-QqQ-MS/MS Technique

**Figure 1:**
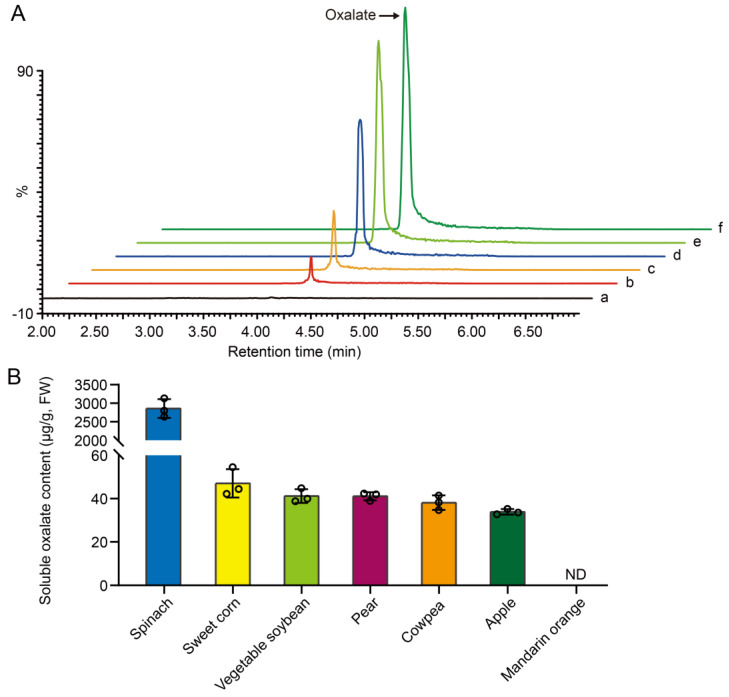
Quantification of oxalate via UPLC-QqQ-MS/MS method. (**A**) Total ion chromatograms (TICs) of oxalate. UPLC-QqQ-MS/MS analysis of oxalate standard solutions at various concentrations. a, blank injection; b–f, different concentrations of oxalate standard solutions, with concentrations of: 2 ppm, 4 ppm, 10 ppm, 16 ppm and 20 ppm. (**B**) Oxalate levels in selected vegetables and fruits. ND, not detected. Error bars indicate ± SD from three biological repeated samples.

Following optimization, we developed a new method for oxalate detection. We tested multiple concentrations of oxalate standard solutions and generated corresponding total ion chromatograms. The retention times for the oxalate standards across five concentrations consistently occurred at approximately 4.10 min (Figure S1 and Figure 1A). To assess the method’s applicability, we measured soluble oxalate levels in various commonly available vegetables and fruits, including spinach (*Spinacia oleracea*), sweet corn (*Zea mays* var. *rugosa*), vegetable soybean (*Glycine max*), immature soybeans, pear (*Pyrus* spp.), cowpea (*Vigna unguiculata*), apple (*Malus pumila*), and mandarin orange (*Citrus reticulata*). Oxalate was undetectable in mandarin orange, while varying concentrations were identified in the other produce (Figure 1B).

We next validated the UPLC-QqQ-MS/MS detection of oxalate levels in maize kernels. Analytical curves were generated using five different concentration points for oxalates. The key parameters such as the linearity, correlation coefficient (r), limit of detection (LOD), and limit of quantification (LOQ) are provided in Table S1. The results indicate strong linearity across the concentration range tested (*r* = 0.99906). Taking into account additional significant validation parameters, the newly developed UPLC-QqQ-MS/MS method demonstrated recovery rates between 93.61% and 107.37%, accompanied by a relative standard deviation (RSD) of less than 14.8% (see Table S2). Regarding precision and accuracy, three independent replicates yielded RSD values ranging from 4.94% to 19.99%, while the absolute relative error (RE) remained consistently under 9.45% (Table S3). Collectively, our data confirmed that the oxalate content in sweet corn is higher compared to certain fruits and vegetables (Figure 1B). Thus, reducing the oxalate content in corn is advantageous for dietary health.

### 2.2. O7 Overexpression Reduces Oxalate Levels in Maize Kernels

**Figure 2:**
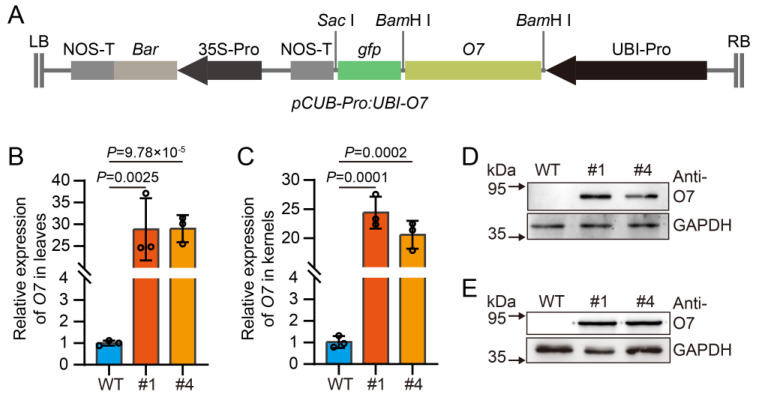
Identification of *O7* overexpression lines. (**A**) Schematic diagram of pCUB-Pro: UBI-*O7*-gfp. (**B**,**C**) Quantification of relative *O7* expression in leaves (**B**) and kernels (**C**) of *O7* overexpression lines (#1, #4). (**D**,**E**) Protein levels assessment in leaves (**D**) and kernels (**E**) of *O7* overexpression lines (#1, #4). GAPDH antibody used as the loading control. Error bars indicate ± SD from three biological repeated samples. Statistical significance was determined using A two-tailed Student’s *t*-test.

The oxalyl-CoA synthetase, encoded by the *O7* gene, initiates the pathway responsible for oxalate acetylation degradation by catalyzing the conversion of oxalate into oxalyl-CoA [30]. A loss of function in this enzyme results in the excessive accumulation of oxalate in seeds. To determine whether overexpressing *O7* can decrease oxalate levels in maize kernels, the *O7* overexpression vector was used to transform the inbred line KN5585 [31] (Figure 2A). A total of 10 independent transgenic events were obtained. Subsequently, RT-qPCR and Western Blot analyses were conducted to detect transgenic events, and two independent T_2_ overexpressed lines (named #1 and #4) were identified (Figure S2 and Figure 2B–E).

**Figure 3:**
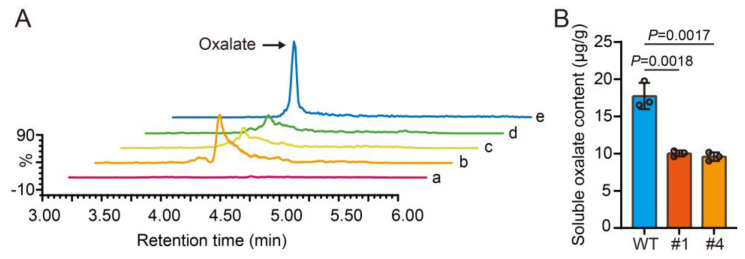
Analysis of oxalate content in kernels from WT and *O7* overexpression lines. (**A**) Total ion chromatograms (TICs) of oxalate. a, blank injection; b, kernels from WT; c and d, kernels from *O7* overexpression lines (#1 and #4, respectively); e, oxalate standard solution with concentration of 10 ppm. (**B**) Quantitative representation of UPLC-QqQ-MS/MS analysis results for soluble oxalate content in maize kernels. Error bars represent ± SD from three biological repeated samples. Statistical significance was assessed using a two-tailed Student’s *t*-test.

Next, we employed a UPLC-QqQ-MS/MS method to measure soluble oxalate levels in maize kernels. Our results revealed that the oxalate content in the kernels of both *O7* overexpression lines (10.02 μg/g in #1, 9.59 μg/g in #4) decreased by approximately 43% compared to the WT (17.75 μg/g) (Figure S3 and Figure 3A,B). The overexpression of oxalate oxidase can reduce oxalate levels.

### 2.3. O7 Overexpression of Increases Specific Micronutrients in Seeds

**Figure 4:**
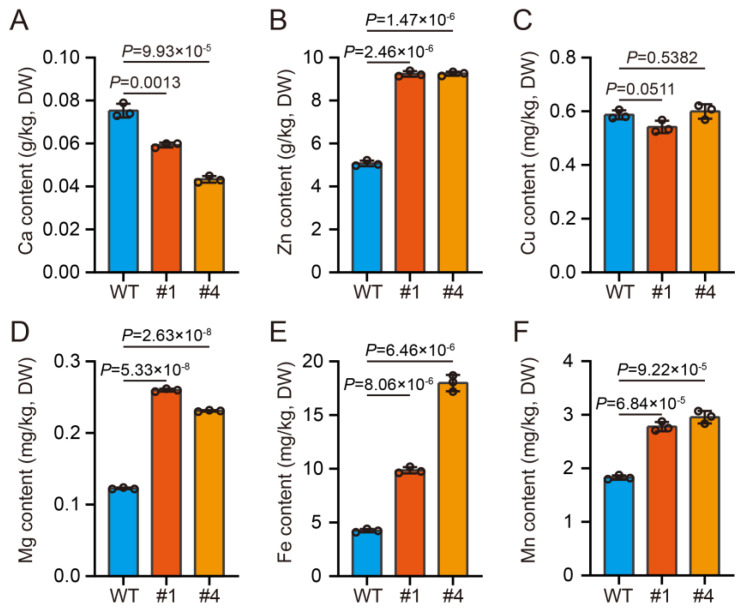
Evaluation of micronutrients in kernels. (**A**–**F**) Concentrations of calcium (**A**), zinc (**B**), copper (**C**), magnesium (**D**), iron (**E**), and manganese (**F**) measured using ICP-OES in seeds of WT and *O7* overexpression lines (#1, #4). Error bars represent the ± SD from three biological repeated samples. Statistical significance was determined using a two-tailed Student’s *t*-test.

Oxalate has a strong affinity for metal ions. The overexpression of oxalate decarboxylase from *Flammulina velutipes* has been shown to significantly reduce oxalate levels and increase micronutrients content in soybean and grass pea seeds [32]. Consequently, we explored whether the overexpression of *O7* can elevate micronutrient levels in maize kernels. We performed a content analysis of calcium, zinc, copper, magnesium, iron, and manganese using ICP-OES. The results revealed a significant increase in zinc, magnesium, iron, and manganese levels compared to the WT, although calcium and copper levels showed no significant change (Table S2; Figure 4). These findings indicate that reducing oxalate content in kernels can enhance the accumulation of micronutrients.

### 2.4. Kernel Development and Storage Substances Remain Unaffected in O7 Overexpression Lines

**Figure 5:**
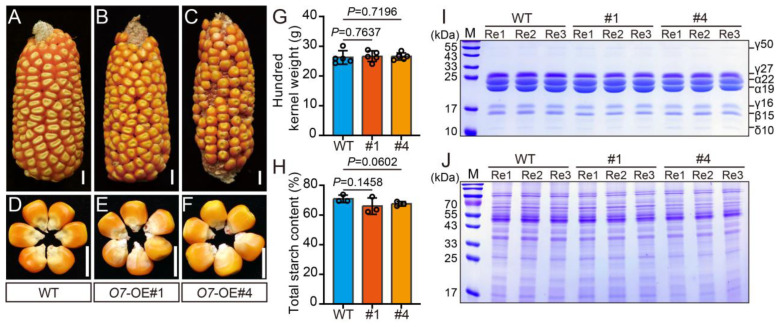
Phenotypic characterization of *O7* overexpression seeds. (**A**–**C**) Mature ears of WT (**A**) and *O7* overexpression lines (#1, #4) (**B**,**C**), bars = 10 mm. (**D**–**F**) Mature kernels of WT (**D**) and *O7* overexpression lines (#1, #4) (**E**,**F**), bars = 10 mm. (**G**) Comparison of hundred kernel weight between WT and *O7* overexpression lines (#1, #4). (**H**) Comparison of total starch content in kernels between WT and *O7* overexpression lines (#1, #4). (**I**,**J**) Zein (**I**) and nonzein (**J**) Content in mature kernels of WT and *O7* overexpression lines (#1, #4). Error bars represent ± SD from five biological repeated samples (**G**) and three biological repeated samples (**H**). A two-tailed Student’s *t*-test was used to determine *p* values.

Compared to the WT, no significant differences were observed in the mature ears of *O7* overexpression lines, and there were no apparent changes in the visual quality of the kernels (Figure 5A–F). Additionally, we evaluated the hundred-kernel weight of maize kernels and found that, consistent with the quality of ears and kernels, there were no significant changes in this measurement (Figure 5G). These findings collectively indicate that the overexpression of *O7* does not affect the development and visual quality of maize ears.

We also assessed the total starch content in mature kernels. The results showed no significant difference in total starch content between the *O7* overexpression lines and the WT, with the total starch content remaining at approximately 70% (Figure 5H).

Previous studies have shown that o7 mutants exhibited decreased zein levels [33]. To explore the effect of *O7* on protein accumulation, we extracted proteins from the endosperm of overexpression lines for analysis. SDS-PAGE results revealed no noticeable differences in either zein or nonzein between the *O7* overexpression lines and the WT, indicating that *O7* overexpression does not affect protein accumulation in seeds (Figure 5I,J).

### 2.5. Overexpression of Ocd1 Does Not Affect Oxalate Content in Maize Kernels

Previous research on *O7* activity indicated that the production of oxalyl-CoA is associated with oxalate concentration [30]. In this study, overexpression of *O7* led to decreased oxalate levels in kernels. Consequently, the reduction in oxalate levels due to *O7* overexpression likely leads to an increase in the downstream product oxalyl-CoA. This elevated oxalyl-CoA, possibly serving as a substrate, subsequently induces the upregulation of *Ocd1* expression. Our results have confirmed this hypothesis (Figure S6A).

**Figure 6:**
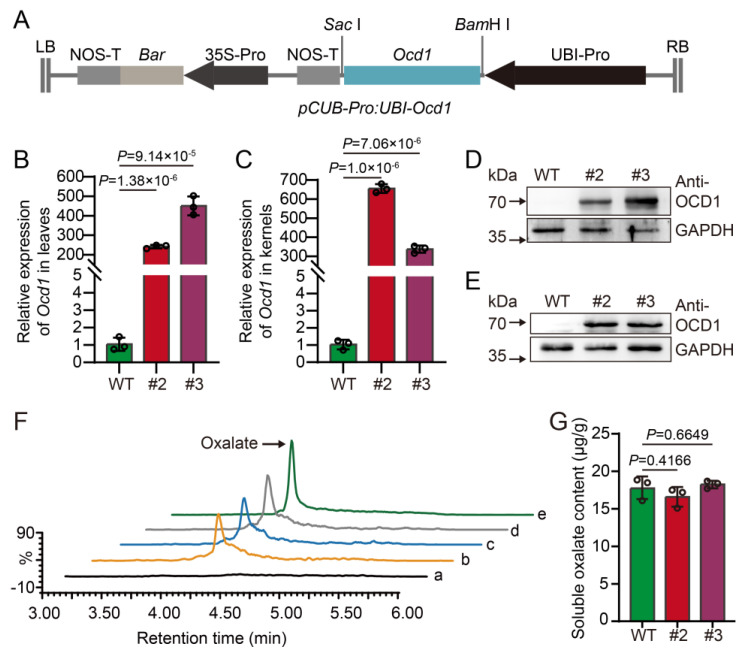
Overexpression of *Ocd1* does not impact oxalate content in maize kernels (**A**) Schematic representation of pCUB-Pro: UBI-*Ocd1*. (**B**,**C**) Quantification of relative *Ocd1* expression of in leaves (**B**) and kernels (**C**) of *Ocd1* overexpression lines (#2, #3). (**D**,**E**) Detection of protein levels in leaves (**D**) and kernels (**E**) of *Ocd1* overexpression lines. GAPDH antibody used as the loading control. (**F**) Total ion chromatograms (TICs) of oxalate. a, blank injection; b, kernels from WT; c and d, kernels from *Ocd1* overexpression lines (#2, #3); e, oxalate standard solution with concentration of 10 ppm. (**G**) Quantitative representation of UPLC-QqQ-MS/MS analysis results for soluble oxalate content in maize kernels. Error bars represent the ± SD from three biological repeated samples. A two-tailed Student’s *t*-test was used to determine *p* values.

*Ocd1* encodes oxalyl-CoA decarboxylase, which catalyzes the breakdown of oxalyl-CoA into formyl-CoA and CO_2_. The impairment of OCD1 function may result in heightened oxalate accumulation within the kernels. However, compared to the WT, the overexpression of *Ocd1* does not reduce the oxalate content in kernels (Figures S4 and S5 and Figure 6). Then, we examined the relative expression levels of *O7* in kernels of *Ocd1* overexpression lines and found no significant changes in the *O7* gene (Figure S6B).

## 3. Discussion

The UPLC-QqQ-MS/MS method, combined with a HILIC column, offers a sensitive and precise approach for quantifying oxalate in biological samples. Unlike traditional C_18_ columns, which pose a risk of precipitation, our technique avoids such issues, ensuring equipment safety and reliable results. The method demonstrated consistent retention times for oxalate across varying concentrations and a high coefficient of determination, confirming its accuracy. When applied to various vegetables and fruits, the study identified significant differences in oxalate levels, with spinach exhibiting the highest concentrations, consistent with previous research [1]. Sweet corn was also found to have a relatively high oxalate content, suggesting that reducing oxalate levels in corn could be beneficial for health. This method not only enhances the accuracy of oxalate measurement but also provides valuable insights for dietary management.

Oxalyl-CoA synthetase is a conserved key enzyme for oxalate degradation in multiple species [28,30,34,35,36,37]. This study further confirmed the significant effect of oxalyl-CoA synthetase in reducing oxalate levels in crops, providing an important candidate gene for reducing oxalate content in high-oxalate vegetables such as spinach. Some high-oxalate vegetables can have most of their oxalate removed during cooking. Corn is covered with a thick pericarp, which may affect the removal of oxalate. Additionally, since sweet corn can be consumed directly, using genetic engineering to reduce the oxalate content in the kernels can decrease intake, thereby promoting health.

Oxalate also plays a crucial role in the pathogenicity of pathogenic fungi, aiding in nutrient acquisition, regulating toxin production, and contributing to lignin degradation [38]. It is essential to investigate whether the low-oxalate corn can be more resistant to pests and diseases in the future.

The observed increase in metal ion levels following the reduction in oxalate content in maize kernels can be attributed to the strong chelating properties of oxalate [39]. This chelation process can limit the accumulation of essential micronutrients, such as zinc, magnesium, iron, and manganese, within plant tissues. Specifically, the significant increases in zinc, magnesium, iron, and manganese levels observed in the *O7* overexpression lines suggest that the reduction in oxalate allowed for greater accumulation of these essential nutrients. Conversely, the levels of calcium and copper did not show significant changes, which could be attributed to different interactions between these metals and oxalate, or possibly their different uptake and accumulation mechanisms in maize. These findings highlight the potential for manipulating oxalate levels in crops to improve the nutritional quality of food. By reducing oxalate content, it may be possible to enhance the bioavailability of essential micronutrients, which could be beneficial for both plant health and human nutrition.

The analysis of these maize kernels’ phenotype revealed that lower oxalate in *O7* overexpression lines does not significantly impact several key quality attributes. Despite observing no significant differences in the visual quality compared to the wild type, it is notable that the hundred-kernel weight and total starch content also remained unaffected. This suggests that the *O7* gene, while influencing other aspects of maize physiology, does not alter these fundamental characteristics of kernel development and composition. The lack of impact on protein composition and starch content in our study suggests that O7’s role might be more specific to other biochemical pathways or processes not directly related to these metrics. Consequently, overexpressing *O7* can enhance micronutrient content without compromising kernel characteristics, suggesting its potential for improving nutritional quality in maize.

In the oxalate acetylation pathway, O7, as the first enzyme, can directly degrade oxalate, resulting in a marked decrease in oxalate content, whereas OCD1, as the subsequent enzyme, relies on the products of the preceding enzymatic step for oxalate degradation [30]. Hence, even with OCD1 overaccumulation, its effectiveness in oxalate degradation remains limited.

## 4. Materials and Methods

### 4.1. Plant Growth Conditions

All genetically modified maize lines were produced by WIMI Bio (Changzhou, China) within the KN5585 genetic background. The wild type (WT) utilized in this research was derived from a non-mutant sibling of transgenic plants. For experimental purposes, both WT and *O7*-OE transgenic lines were grown in Hefei (31° N, 117° E), Anhui Province, China during the summer season and in Sanya (18° N, 109° E), Hainan Province, China during the winter season. The row and plant spacing was maintained at 60 cm and 15 cm, respectively.

### 4.2. Plasmid Construction and Positive Lines Screening

To construct the *pCUB-Pro: UBI-O7-gfp* vector for transformation, the coding region of *O7* was inserted into the *pCUB-Pro: UBI-gfp* vector via the *Bam*H I restriction sites. For generating the *O7* overexpression transgenic maize lines, the *Agrobacterium tumefaciens* strain EHA105 that harbors the *pCUB-Pro: UBI-O7-gfp* construct was utilized to transform the inbred line KN5585. Positive transgenic lines from various events were selected by spraying with a Basta solution (0.1%, *v*/*v*). Two independent high-expression transgenic lines were confirmed by Real-Time PCR (RT-qPCR) analysis and Western Blot analysis and selected for further investigation. The primers used in these identification steps are detailed in Table S1.

### 4.3. RNA Extraction and RT-qPCR

Total RNA of seedling leaves and 20 days after pollination (DAP) kernels were extracted using the plant total RNA isolation kit (RC401, Vazyme, Nanjing, China). For RT-qPCR, 1 μg of total RNA was reverse transcribed into first-strand complementary DNA (cDNA) using the All-in-one RT super-mix perfect for qPCR (R333-01, Vazyme, Nanjing) according to the manufacturer’s instructions. RT-qPCR was performed with SYBR Green master mix (Q111, Vazyme, Nanjing, China) on a Real-Time PCR System, (Thermo Scientific, Waltham, MA, USA) following the manufacturer’s protocol. The *Ubi* gene was used as an internal control for RT-qPCR. Each sample was analyzed in three biological replicates. All primer sequences for RT-qPCR are listed in Table S1. 

### 4.4. Total Protein Extraction and Western Blot

Total protein was extracted from seedling leaves and 20 DAP kernels of wild-type, *O7*-OE, and *Ocd1*-OE with the lysis buffer (90 mM HEPES, pH 8.0, 1 mM EDTA, pH 8.0, 10% [*v*/*v*] glycerol, and 5 mM DTT). Briefly, 1 g of seedling leaves or 0.5 g of 20 DAP kernels were collected and rapidly frozen in liquid nitrogen and ground into a fine powder. An amount of 300 μL of lysis buffer was added for complete dissolution, followed by a 2 h incubation on ice with continuous inversion to ensure uniform mixing. The mixture was centrifuged at 13,000 rpm for 15 min at 4 °C, and 200 μL of the supernatant was transferred to a fresh tube. Protein concentration was determined using the Bradford assay and normalized to achieve consistency. Subsequently, SDS-PAGE Sample Loading Buffer was added, followed by thorough mixing. The samples were then prepared with SDS-PAGE loading buffer and heated at 95 °C for 5 min to fully denature the proteins. 

Each sample contained 20 μg total protein and were separated on a 12% (*w*/*v*) SDS-PAGE gel and then transferred onto a hydrophobic PVDF transfer membrane (Merck KGaA, Darmstadt, Germany). Immunoblot analysis was conducted as described previously [40], using antibodies against O7 and OCD1 at dilutions of 1:5000 and 1:2000, respectively. The GAPDH antibody (60004-1-Ig, Proteintech, Wuhan, China) was used as a loading control at a 1:10,000 dilution. Secondary antibodies included goat anti-rabbit IgG (H+L) and goat anti-mouse IgG (H+L) (HS101-01, HS201-01, Transgen, Beijing) diluted at 1:5000 for the immunoreaction. Signal detection was performed using the ECL Western Blot substrate (180-506, Tanon, Shanghai, China) and visualization was performed with the Tanon-5200 imaging system (Tanon, Shanghai, China).

### 4.5. Sample Preparation for Oxalate Measurement

To measure oxalate levels in maize, or certain fruits and vegetables, 20 DAP maize kernels, fruits, and vegetables (500 mg/FW) were homogenized, and oxalate was extracted using methanol/water 50/50 (*v*/*v*) (Sigma, St. Louis, MO, USA) with ultrasonication [41,42]. The extract underwent 15 min of ultrasonication with intermittent shaking for thorough mixing, followed by centrifugation at 12,000 rpm for 10 min at 4 °C. The supernatant was transferred to new centrifuge tubes, and 1 mL of 50% methanol/water (*v*/*v*) was added to the pellet, followed by vortexing and repetition of the previous steps. The crude oxalate extract was filtered through a 0.22 μm pore size filter, and the filtrate was stored at 4 °C.

### 4.6. UPLC-QqQ-MS/MS Analysis

Oxalate quantification was performed using UPLC-QqQ-MS/MS analysis. Chromatographic separation was conducted using an ultra-high-performance liquid chromatography system (ACQUITY UPLC I-Class) with an ACQUITY UPLC BEH amide column (130 Å, 1.7 µm, 2.1 mm × 100 mm, Waters, Milford, MA, USA). Quantitative analysis was performed using Xevo TQ-S (Waters, Milford, MA, USA).

To optimize oxalate detection, a 1000 ppb oxalate solution in 50% methanol/water (*v*/*v*) was introduced into the ion source at a flow rate of 10 μL/min under negative ionization mode. Oxalate exhibited strong signal sensitivity as [M-H]^−^ ions at *m*/*z* 88.9 in the Parent Ion Scan mode. Consequently, the MS/MS spectra presented abundant product ions corresponding to the transition *m*/*z* 88.9 → 61.0 in the Daughter Ion Scan mode. The cone voltage and collision energy were optimized. They were set to 30 V and 6 V, respectively, to achieve the best signal intensity. This process allowed for the selection of the most abundant, specific, and stable MRM channels in MRM mode.

After systematic assessment, the final optimal conditions were determined as a mobile phase A composed of acetonitrile/water 50/50 (*v*/*v*) with 20 mM ammonium acetate and acetonitrile/water 85/15 (*v*/*v*) with 20 mM ammonium acetate. Separation was achieved with a flow rate of 0.4 mL/min using the following elution program: 0–1.20 min, column equilibration (0.1% A); 1.20–2.20 min, gradient to 70% A; 2.20–2.80 min, gradient to 99.9% A; 2.80–4.00 min, isocratic hold at 99.9% A; 4.00–4.01 min, gradient to 0.1% A; and 4.01–7 min, isocratic hold at 0.1% A. The injection volume was 5 μL, and the column temperature was maintained at 50 °C. All samples were stored at 4 °C during analysis.

The ion source operational parameters were set as follows: the ion source temperature was held at 150 °C, the capillary voltage was set to 3.0 kV, and the desolation temperature was maintained at 400 °C. The cone gas (nitrogen) flow rate was set at 800 L/h.

### 4.7. Validation of UPLC-QqQ-MS/MS Analytical Method

The UPLC-QqQ-MS/MS method was validated in terms of linearity, accuracy, precision, and sensitivity, adhering to established analytical method guidelines [43]. Accuracy and precision were assessed using three key metrics: the relative standard deviation (RSD) for oxalate recovery, RSD from triplicate trials, and the relative error (RE). These parameters were evaluated by fortifying maize grain samples with standard oxalate solutions at three distinct concentration levels. Quantification of oxalate was carried out through three separate experimental runs. Sensitivity was also determined by calculating the limit of detection (LOD) and limit of quantification (LOQ), which corresponded to the lowest spiked concentrations that produced signal-to-noise ratios (S/N) greater than 3 and 10, respectively.

### 4.8. Determination of Micronutrients

Micronutrient levels in maize seeds were analyzed using inductively coupled plasma optical emission spectroscopy (ICP-OES). Mature seeds from different ears are ground into powder and dried. Then, 0.4 g of dried seed powder from each sample was weighed, digested with nitric acid, and analyzed for total calcium, zinc, copper, magnesium, iron, and manganese content using ICP-OES (Table S2).

### 4.9. Protein and Starch Content Measurement

To compare protein content, mature WT and transgenic seeds were ground into fine flour using steel balls. Each sample was analyzed in three biological replicates. An amount of 100 mg of flour was extracted for zein and nonzein protein analysis. For zein extraction, 100 mg of flour was mixed with 1 mL of zein extraction buffer (composed of 70% ethanol, 2% β-mercaptoethanol [*v*/*v*], 3.75 mmol sodium borate, pH 10, and 0.3% SDS). For nonzein extraction, the flour precipitate was subjected to three additional extractions with zein buffer to eliminate excess zein. SDS-PAGE analysis was conducted to evaluate the accumulation of zein and nonzein proteins in both wild-type and transgenic maize [40].

The above obtained fine flours were filtered through an 80-mesh nylon sieve and dried overnight at 42 °C for starch content determination. Total starch was measured using the Megazyme assay kit (K-TSTA, Megazyme, Lansing, MI, USA) according to the manufacturer’s instructions.

### 4.10. Statistical Analysis

All UPLC-QqQ-MS/MS detection data were recorded and processed using MassLynx software (version 4.2, Waters corporation, Milford, MA, USA). Experimental data were analyzed by GraphPad Prism (version 8.4.3, GraphPad Software, Boston, MA, USA). Statistical significance of the experimental data was evaluated using Student’s *t*-tests and Duncan’s test (Microsoft Excel 2019 version 2405).

### 4.11. Accession Numbers

The sequence data from this study are available in MaizeGDB (https://www.maizegdb.org/) under accession numbers Zm00001d008739 (*Ocd1*), Zm00001d026649 (*O7*), and Zm00001d015327 (*Ubi*).

## 5. Conclusions

In this study, we developed a high-throughput and highly sensitive method for detecting oxalate using UPLC-QqQ-MS/MS, significantly improving the accuracy of oxalate measurement in food products. Furthermore, we successfully overexpressed the *O7* gene, which encodes oxalyl-CoA synthetase, a key enzyme in the maize oxalate degradation pathway, resulting in the creation of *O7*-OE maize lines. The analysis using the UPLC-QqQ-MS/MS method showed a reduction of approximately 43% in oxalate content in the kernels of these transgenic lines, along with an increase in certain micronutrients, such as zinc. Importantly, these transgenic maize lines maintained normal levels of seed storage compounds and preserved other essential agronomic traits. Our findings highlight the developed detection method as a major advancement in oxalate quantification. Additionally, the generation of low-oxalate maize germplasm presents a promising strategy for improving public health by offering maize with enhanced nutritional quality.

## Data Availability

The original contributions presented in the study are included in the article and Supplementary Materials; further inquiries can be directed to the corresponding authors.

## Supplementary Materials

The following supporting information can be downloaded at: https://www.mdpi.com/article/10.3390/plants13212950/s1, Figure S1: Total ion chromatograms (TICs) of oxalate standard solutions; Figure S2: Screening of O7 overexpression transgenic lines; Figure S3: Total ion chromatograms (TICs) of oxalate in kernels from wild type (A), O7-OE#1 (B) and O7-OE#4 (C), respectively; Figure S4: Screening of Ocd1 overexpressed transgenic lines; Figure S5: Total ion chromatograms (TICs) of oxalate in kernels from wild type (A), Ocd1-OE#2 (B) and Ocd1-OE#3 (C), respectively; Figure S6: (A) The relative expression level of Ocd1 in kernels of O7 overexpressed lines (#1, #4). (B) The relative expression level of O7 in kernels of Ocd1 overexpressed lines (#2, #3). Table S1: Linearity, correlation coefficient (r), limits of detection (LOD) and quantification (LOQ) of oxalate by UPLC-QqQ-MRM; Table S2: Recovery rates of oxalate by UPLC-QqQ-MRM; Table S3: Precision and accuracy of oxalate by UPLC-QQQ-MRM; Table S4: Primers used in this study; Table S5: Determining micronutrient concentrations in maize seeds using ICP-OES.

## References

[1] Savage G.P., Vanhanen L., Mason S.M., Ross A.B. (2000). Effect of Cooking on the Soluble and Insoluble Oxalate Content of Some New Zealand Foods. J. Food Compos. Anal..

[2] Thomas E., von Unruh G.E., Hesse A. (2008). Influence of a low- and a high-oxalate vegetarian diet on intestinal oxalate absorption and urinary excretion. Eur. J. Clin. Nutr..

[3] Voss S., Hesse A., Zimmermann D.J., Sauerbruch T., von Unruh G.E. (2006). Intestinal oxalate absorption is higher in idiopathic calcium oxalate stone formers than in healthy controls: Measurements with the [^13^C_2_]oxalate absorption test. J. Urol..

[4] Worcester E.M., Coe F.L. (2010). Clinical practice. Calcium kidney stones. N. Engl. J. Med..

[5] Wang P., Li Z., Li H., Zhang D., Wang W., Xu X., Xie Q., Duan Z., Xia X., Guo G. (2024). SMART CROPs. New Crops.

[6] Ruan Q.-Y., Zheng X.-Q., Chen B.-L., Xiao Y., Peng X.-X., Leung D.W.M., Liu E.E. (2013). Determination of total oxalate contents of a great variety of foods commonly available in Southern China using an oxalate oxidase prepared from wheat bran. J. Food Compos. Anal..

[7] Mou B. (2008). Evaluation of Oxalate Concentration in the U.S. Spinach Germplasm Collection. HortScience.

[8] Arias-Rico J., Macías-León F.J., Alanís-García E., Cruz-Cansino N.D.S., Jaramillo-Morales O.A., Barrera-Gálvez R., Ramírez-Moreno E. (2020). Study of Edible Plants: Effects of Boiling on Nutritional, Antioxidant, and Physicochemical Properties. Foods.

[9] Siener R., Seidler A., HÖNow R. (2021). Oxalate-rich foods. Food Sci. Technol..

[10] Masár M., Žúborová M., Kaniansky D., Stanislawski B. (2003). Determination of oxalate in beer by zone electrophoresis on a chip with conductivity detection. J. Sep. Sci..

[11] March J.G., Simonet B.M., Gráses F., Muñoz J.A., Valiente M. (2003). Determination of trace amounts of oxalate in renal calculi and related samples by gas chromatography-mass spectrometry. Chromatographia.

[12] Pundir C.S., Chauhan N., Rajneesh, Verma M., Ravi (2011). A novel amperometric biosensor for oxalate determination using multi-walled carbon nanotube-gold nanoparticle composite. Sens. Actuators B Chem..

[13] Maya F., Estela J.M., Cerdà V. (2011). Multisyringe ion chromatography with chemiluminescence detection for the determination of oxalate in beer and urine samples. Microchim. Acta.

[14] Li H., Liu Y., Zhang Q., Zhan H. (2014). Determination of the oxalate content in food by headspace gas chromatography. Anal. Methods.

[15] Hönow R., Hesse A. (2002). Comparison of extraction methods for the determination of soluble and total oxalate in foods by HPLC-enzyme-reactor. Food Chem..

[16] Judprasong K., Charoenkiatkul S., Sungpuag P., Vasanachitt K., Nakjamanong Y. (2006). Total and soluble oxalate contents in Thai vegetables, cereal grains and legume seeds and their changes after cooking. J. Food Compos. Anal..

[17] Libert B. (1981). Rapid determination of oxalic acid by reversed-phase high-performance liquid chromatography. J. Chromatogr. A.

[18] Valentão P., Lopes G., Valente M., Barbosa P., Andrade P.B., Silva B.M., Baptista P., Seabra R.M. (2005). Quantitation of nine organic acids in wild mushrooms. J. Agric. Food Chem..

[19] Naidong W. (2003). Bioanalytical liquid chromatography tandem mass spectrometry methods on underivatized silica columns with aqueous/organic mobile phases. J. Chromatogr. B.

[20] Hemström P., Irgum K. (2006). Hydrophilic interaction chromatography. J. Sep. Sci..

[21] Cubbon S., Bradbury T., Wilson J., Thomas-Oates J. (2007). Hydrophilic interaction chromatography for mass spectrometric metabonomic studies of urine. Anal. Chem..

[22] Spagou K., Tsoukali H., Raikos N., Gika H., Wilson I.D., Theodoridis G. (2010). Hydrophilic interaction chromatography coupled to MS for metabonomic/metabolomic studies. J. Sep. Sci..

[23] Jiao Y., Chen D., Fan M., Quek S.Y. (2019). UPLC-QqQ-MS/MS-based phenolic quantification and antioxidant activity assessment for thinned young kiwifruits. Food Chem..

[24] Sun Y., Li H., Hu J., Li J., Fan Y.W., Liu X.R., Deng Z.Y. (2013). Qualitative and quantitative analysis of phenolics in Tetrastigma hemsleyanum and their antioxidant and antiproliferative activities. J. Agric. Food Chem..

[25] Dong X., Ji R., Guo X., Foster S.J., Chen H., Dong C., Liu Y., Hu Q., Liu S. (2008). Expressing a gene encoding wheat oxalate oxidase enhances resistance to Sclerotinia sclerotiorum in oilseed rape (*Brassica napus*). Planta.

[26] Hu X., Bidney D.L., Yalpani N., Duvick J.P., Crasta O., Folkerts O., Lu G. (2003). Overexpression of a gene encoding hydrogen peroxide-generating oxalate oxidase evokes defense responses in sunflower. Plant Physiol..

[27] Wei F., Hu J., Yang Y., Hao Z.-d., Wu R.-h., Tian B.-m., Cao G.-q., Zang X. (2015). Transgenic Arabidopsis thaliana expressing a wheat oxalate oxidase exhibits hydrogen peroxide related defense response. J. Integr. Agric..

[28] Foster J., Kim H.U., Nakata P.A., Browse J. (2012). A Previously Unknown Oxalyl-CoA Synthetase Is Important for Oxalate Catabolism in Arabidopsis. Plant Cell.

[29] Miclaus M., Wu Y., Xu J.H., Dooner H.K., Messing J. (2011). The maize high-lysine mutant opaque7 is defective in an acyl-CoA synthetase-like protein. Genetics.

[30] Yang J., Fu M., Ji C., Huang Y., Wu Y. (2018). Maize Oxalyl-CoA Decarboxylase1 Degrades Oxalate and Affects the Seed Metabolome and Nutritional Quality. Plant Cell.

[31] Ishida Y., Hiei Y., Komari T. (2007). Agrobacterium-mediated transformation of maize. Nat. Protoc..

[32] Kumar V., Chattopadhyay A., Ghosh S., Irfan M., Chakraborty N., Chakraborty S., Datta A. (2016). Improving nutritional quality and fungal tolerance in soya bean and grass pea by expressing an oxalate decarboxylase. Plant Biotechnol. J..

[33] Misra P.S., Jambunathan R., Mertz E.T., Glover D.V., Barbosa H.M., McWhirter K.S. (1972). Endosperm protein synthesis in maize mutants with increased lysine content. Science.

[34] Lou H.Q., Fan W., Xu J.M., Gong Y.L., Jin J.F., Chen W.W., Liu L.Y., Hai M.R., Yang J.L., Zheng S.J. (2016). An Oxalyl-CoA Synthetase Is Involved in Oxalate Degradation and Aluminum Tolerance. Plant Physiol..

[35] Fan M., Xiao Y., Li M., Chang W. (2016). Crystal Structures of Arabidopsis thaliana Oxalyl-CoA Synthetase Essential for Oxalate Degradation. Mol. Plant.

[36] Xian P., Cai Z., Cheng Y., Lin R., Lian T., Ma Q., Nian H. (2020). Wild Soybean Oxalyl-CoA Synthetase Degrades Oxalate and Affects the Tolerance to Cadmium and Aluminum Stresses. Int. J. Mol. Sci..

[37] Li P., He Q., Jin J., Liu Y., Wen Y., Zhao K., Mao G., Fan W., Yang J. (2022). Tomato Oxalyl-CoA Synthetase Degrades Oxalate and Affects Fruit Quality. Front. Plant Sci..

[38] Foster J., Luo B., Nakata P.A. (2016). An Oxalyl-CoA Dependent Pathway of Oxalate Catabolism Plays a Role in Regulating Calcium Oxalate Crystal Accumulation and Defending against Oxalate-Secreting Phytopathogens in Medicago truncatula. PLoS ONE.

[39] Nakata P.A. (2012). Influence of calcium oxalate crystal accumulation on the calcium content of seeds from Medicago truncatula. Plant Sci..

[40] Wang T., Chang Y., Zhao K., Dong Q., Yang J. (2022). Maize RNA 3′-terminal phosphate cyclase-like protein promotes 18S pre-rRNA cleavage and is important for kernel development. Plant Cell.

[41] Liu X.X., Zhou K., Hu Y., Jin R., Lu L.L., Jin C.W., Lin X.Y. (2015). Oxalate synthesis in leaves is associated with root uptake of nitrate and its assimilation in spinach (*Spinacia oleracea* L.) plants. J. Sci. Food Agric..

[42] Das S.G., Savage G.P. (2013). Oxalate content of Indian spinach dishes cooked in a wok. J. Food Compos. Anal..

[43] Magnusson B., Örnemark U. (2014). Eurachem Guide: The Fitness for Purpose of Analytical Methods—A Laboratory Guide to Method Validation and Related Topics.

