# Supplementary material for: Optimization of Plant Oxalate Quantification and Generation of Low-Oxalate Maize (Zea mays L.) through O7 Overexpression
Source: Plants (Basel). 2024 Oct 22;13(21):2950. doi: 10.3390/plants13212950 (PMC11547668; doi:10.3390/plants13212950)
Supplement: Supplementary file 1 [file plants-13-02950-s001.zip › plants-3233863-supplementary/Supplemental Figure.pdf]

Supplemental Figures

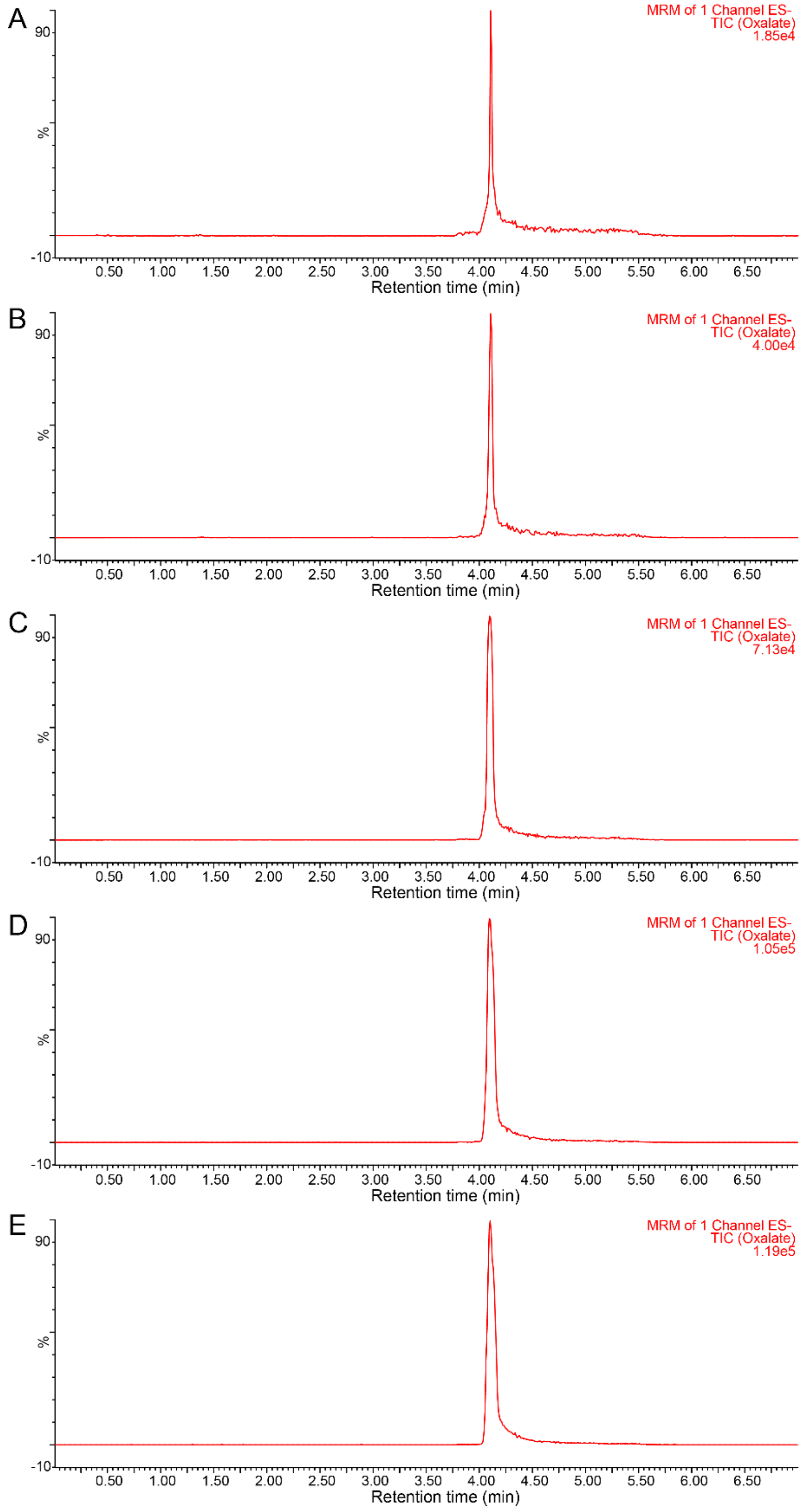

Figure S1. Total ion chromatograms (TICs) of oxalate standard solutions. (A)-(E) Oxalate standard solutions at different concentrations: (A) 2 ppm; (B) 4 ppm; (C) 10 ppm; (D) 16 ppm and (E) 20 ppm.

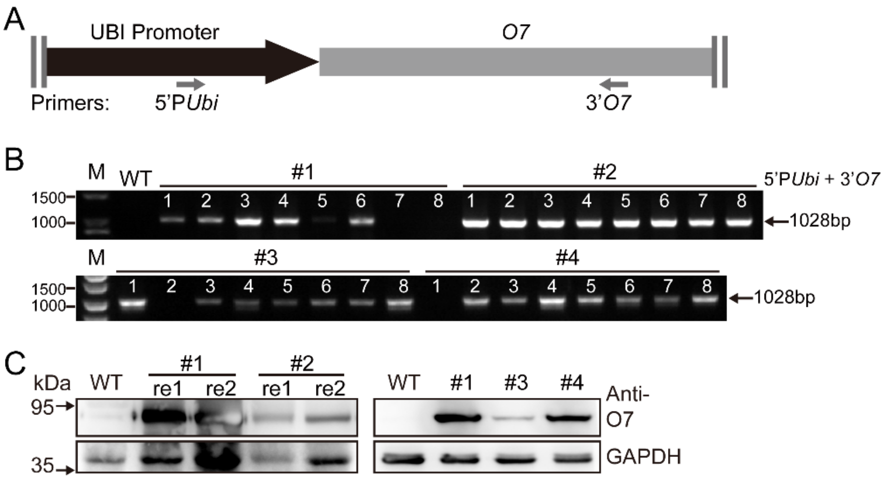

Figure S2. Screening of *O7* overexpression transgenic lines. (A) Diagram illustrating the primers utilized in the identification of positive lines. (B) and (C), Identification of positive lines by PCR (B) and Western blot (C).

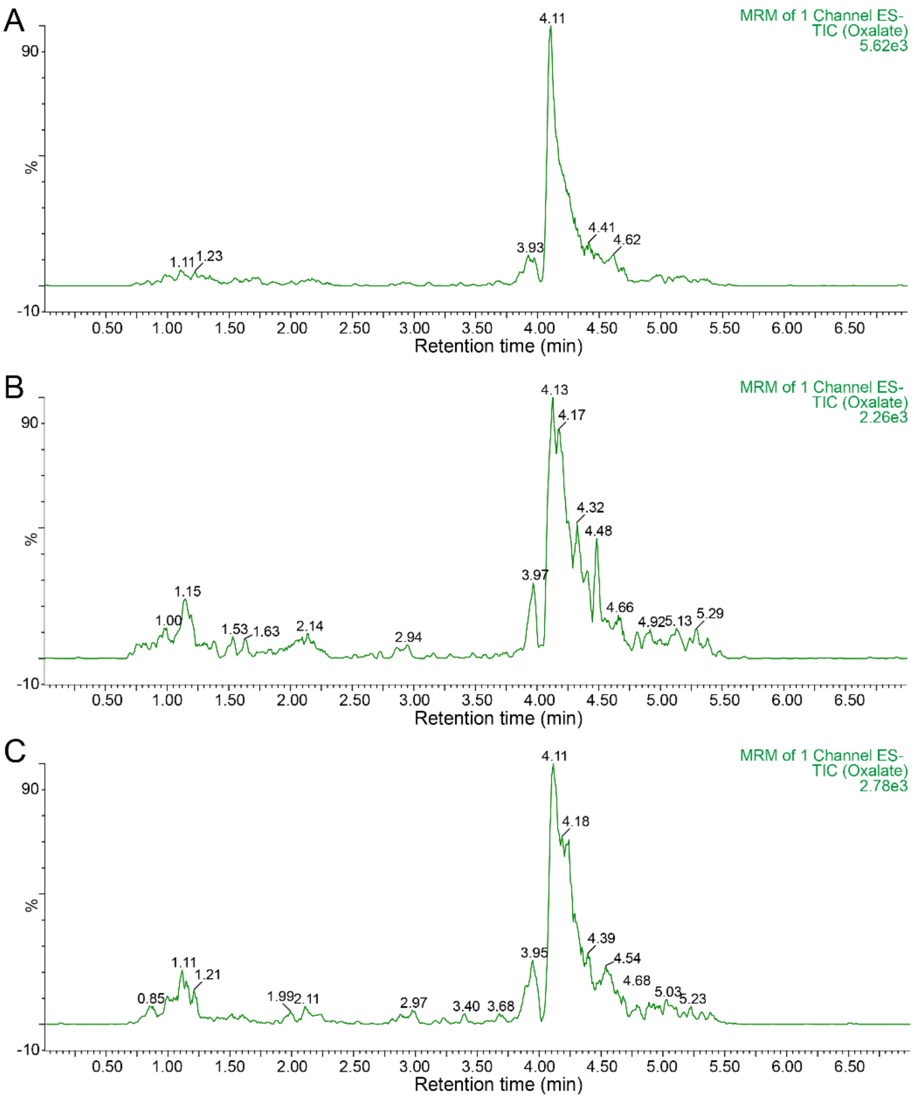

Figure S3. Total ion chromatograms (TICs) of oxalate in kernels from wild-type (A), *O7*-OE#1 (B) and *O7*-OE#4 (C), respectively.

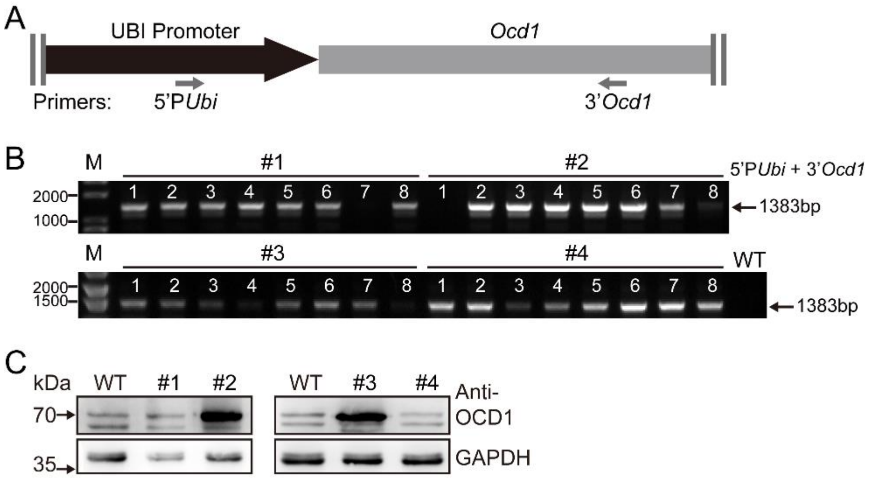

Figure S4. Screening of *Ocd1* overexpressed transgenic lines. (A) Diagram illustrating the primers used in the identification of positive lines. (B) and (C) Identification of positive lines by PCR amplification (B) and Western blot (C).

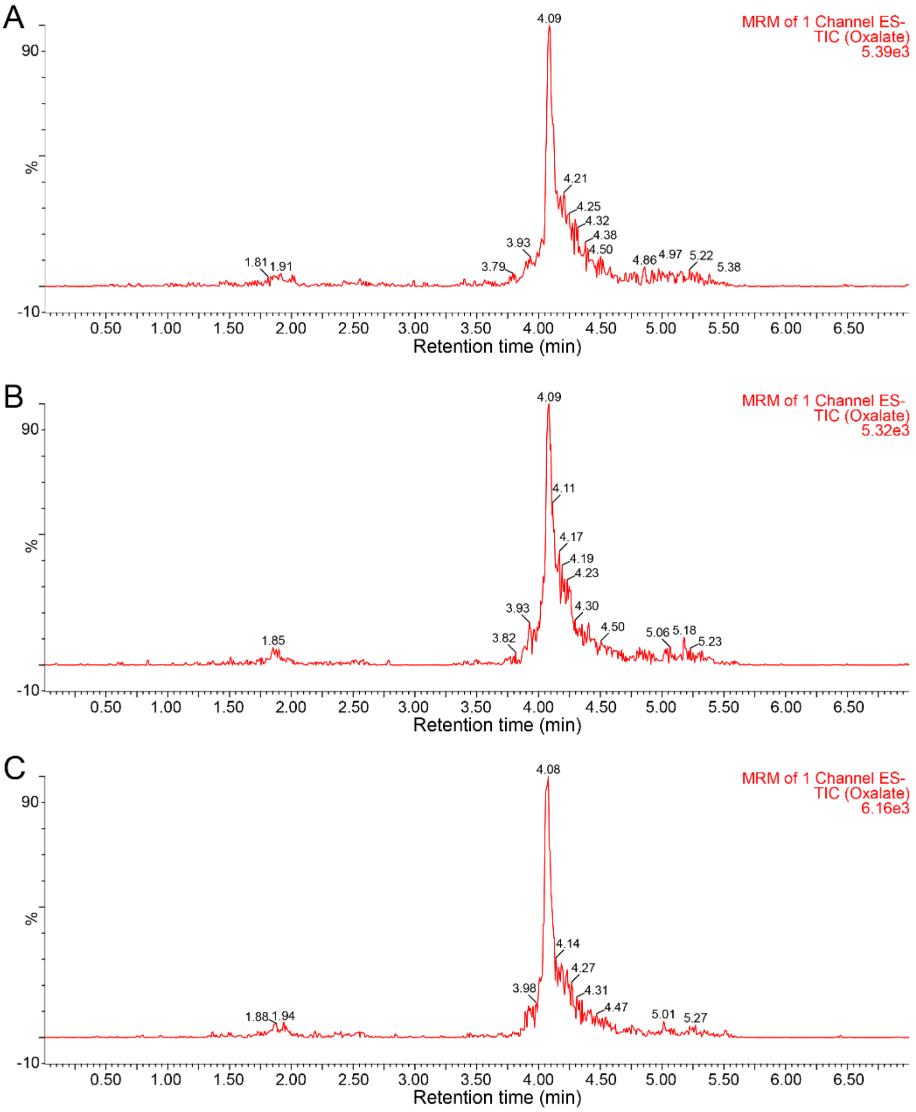

Figure S5. Total ion chromatograms (TICs) of oxalate in kernels from wild-type (A), *Ocd1*-OE#2 (B) and *Ocd1*-OE#3 (C), respectively.

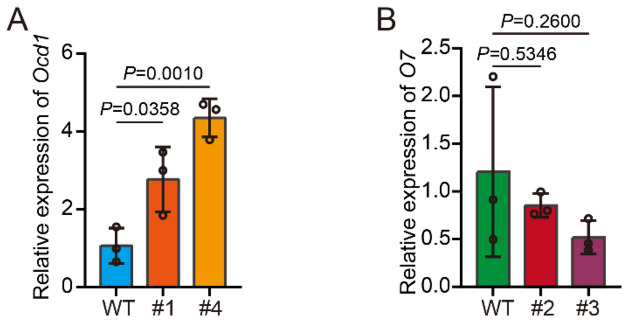

Figure S6. (A) The relative expression level of *Ocd1* in kernels of *O7* overexpressed lines (#1, #4). (B) The relative expression level of *O7* in kernels of *Ocd1* overexpressed lines (#2, #3). Error bars represent the ± SD from three biological repeated samples. A two-tailed Student's *t*-test was used to determine *P* values.
